# Supplementary material for: Interventions Effective in Decreasing Burden in Caregivers of Persons with Dementia: A Meta-Analysis
Source: Nurs Rep. 2024 Apr 15;14(2):931–45. doi: 10.3390/nursrep14020071 (PMC11036232; doi:10.3390/nursrep14020071)
Supplement: Supplementary file 1 [file nursrep-14-00071-s001.zip › nursrep-2889834-supplementary.pdf]

Interventions effective in decreasing burden in caregivers of persons with dementia: A meta-analysis.

Francisco José Rodríguez Alcazar[1*], Raúl Juárez-Vela[2], Juan Luis Sánchez González[3] y Javier Martín-Vallejo[4]

[1] MPhil, Regional Health Management of Castilla y León, Spain; https://orcid.org/0000-0002-7433-7816 ; obifran@hotmail.com
[2] PhD, Group of Research in Care (GRUPAC), Faculty of Health Sciences, University of La Rioja, Logroño, Spain; https://orcid.org/0000-0003-3597-2048
[3] PhD, Department of Physiotherapy, Faculty of Nursing and Physiotherapy, University of Salamanca, Spain; https://orcid.org/0000-0002-4791-6513
[4] PhD, Department of Statistics, Faculty of Medicine, University of Salamanca, Spain; https://orcid.org/0000-0003-3962-7356

* Correspondence: juanluissanchez@usal.es

Table of contents

Supplementary Table S1. Respite care studies. ____ *3*

Supplementary Table S2. Support group studies. ____ *4*

Supplementary Table S3. Individual information studies. ____ *5*

Supplementary Table S4. Workshop studies. ____ *5*

Supplementary Table S5. Group therapy studies. ____ *6*

Supplementary Table S6. Individual therapy studies. ____ *7*

Supplementary Table S7. Jadad scale quality studies. ____ *8*

Supplementary Table S8. Delphi list quality studies. ____ *11*

Supplementary Table S9. Forbes scale quality studies. ____ *13*

Supplementary Table S10. Risk of bias (ROB) 2.0 ____ *15*

Supplementary Table S11. The PRISMA Checklist. ____ *19*

Supplementary Figure S1. Heterogeneity evolution when excluding articles. ____ *23*

Supplementary Figure S2. Heterogeneity evolution when excluding articles. ____ *24*

Supplementary Table S1. Respite care studies.

Author	NCDs	Scale	Intervention time	Intervention end (weeks)	Drop out	Nurse	Country	Relationship	Sex	Education mean (SD)	Age (SD)	Income	Trainer	Control group[c]	Final control group[d]
Quayhangen, 2000 [33]	Combined	(BI)[a]	8	12	UNDEFINED	YES	USA	SPOUSE	0,63	14,42 (0,3)	71,83 (0,8)	NO	COMBINED	WL	SINGLE
Baumgarten, 2002 [56]	Other dementias	(CBI)[b]	12	12	15%	NO	FRANCE	COMBINED	0,72	NO	56,35 (15,84)	NO	UNDEFINED	WL	ALL
Mosello, 2008 [57]	Other dementias	(CBI)[a]	8	8	5%	YES	ITALY	COMBINED	0,65	7,7 (0,7)	57 (2)	NO	COMBINED	CG(UC)	UNDEFINED

Note. [a] Partial data. [b] Global data. [c] Control group without intervention (CG), Control group with alternative intervention (CG-AI), Control group with usual alternative intervention (CG-UC), Waiting list without intervention (WL), Waiting list with alternative intervention (WL-AI), Waiting list with usual alternative intervention (WL-UC). [d] At the end of the study, the control group may or may not receive the new intervention. (CBI)-Caregiver Burden Index; (BI)-Memory and Behavior Problem Checklist;(RMBPC)-Revised Memory and Behavior Problem Checklist;(MBPC)-The Memory and Behavior Problems Checklist;(CSS)-Caregiver Strain Scale;(ICS)-Impact of Caregiver Scale.

Supplementary Table S2. Support group studies.

Author	NCDs	Scale	Intervention time	Intervention end (weeks)	Drop out	Nurse	Country	Relationship	Sex	Education mean (SD)	Age (SD)	Income	Trainer	Control group[c]	Final control group[d]
Zarit, 1987 [45]	Combined	(BI)[b]	8	8	35%	NO	USA	COMBINED	0,63	14,38 (2,69)	62 (12,75)	NO	PSYCHOLOGIST	WL	SINGLE
Hebert, 1994 [58]	Combined	(BI)[b]	8	8	27%	YES	CANADA	SPOUSE	0,63	9,76 (1,56)	61,07 (13,27)	NO	NURSE	CG(UC)	UNDEFINED
Yordi, 1994 [59]	Alzheimer	(BI)[a]	36	36	64%	NO	USA	COMBINED	0,6	NO	63 (14,29)	NO	UNDEFINED	CG(UC)	UNDEFINED
Teri, 1997 [32]	Combined	(BI)[a]	9	9	14%	NO	USA	COMBINED	0,69	14,2 (2,7)	66,9 (11)	NO	THERAPIST	CG(UC); WL	UNDEFINED
Roth, 2005 [60]	Alzheimer	(MBPC)[b]	52	52	23%	NO	USA	SPOUSE	0,58	NO	71,07 (9,08)	NO	SOCIAL WORKER	CG(UC)	UNDEFINED
Chien, 2008 [61]	Combined	(CBI)[b]	24	52	3%	YES	CHINA	COMBINED	0,64	0,68 (NO)	43,9 (9,2)	$18.456	COMBINED	CG(UC)	UNDEFINED
Logsdon, 2011 [62]	Other dementias	(RMBPC)[a]	9	9	4%	NO	USA	COMBINED	0,57	NO	67,94 (13,22)	NO	FACILITATOR	WL	ALL

Note. [a] Partial data. [b] Global data. [c] Control group without intervention (CG), Control group with alternative intervention (CG-AI), Control group with usual alternative intervention (CG-UC), Waiting list without intervention (WL), Waiting list with alternative intervention (WL-AI), Waiting list with usual alternative intervention (WL-UC). [d] At the end of the study, the control group may or may not receive the new intervention. (CBI)-Caregiver Burden Index; (BI)-Memory and Behavior Problem Checklist;(RMBPC)-Revised Memory and Behavior Problem Checklist;(MBPC)-The Memory and Behavior Problems Checklist;(CSS)-Caregiver Strain Scale;(ICS)-Impact of Caregiver Scale.

Supplementary Table S3. Individual information studies.

Author	NCDs	Scale	Intervention time	Intervention end (weeks)	Drop out	Nurse	Country	Relationship	Sex	Education mean (SD)	Age (SD)	Income	Trainer	Control group[c]	Final control group[d]
McCurry, 2005 [28]	Alzheimer	(RMBPC)[a]	8	24	36%	NO	USA	COMBINED	0,72	14,96 (2,47)	63,28 (15,83)	NO	UNDEFINED	CG(AI)	UNDEFINED
Dias, 2008 [29]	Other dementias	(BI)[b]	24	24	27%	NO	INDIA	COMBINED	0,87	NO	54,48 (14,98)	$1.485	PSYCHIATRIST	WL(AI)	UNDEFINED
Gavrilova, 2009 [30]	Alzheimer	(BI)[b]	5	24	12%	NO	RUSSIA	COMBINED	0,73	NO	59,34 (15,54)	NO	UNDEFINED	WL(UC)	UNDEFINED

Note. [a] Partial data. [b] Global data. [c] Control group without intervention (CG), Control group with alternative intervention (CG-AI), Control group with usual alternative intervention (CG-UC), Waiting list without intervention (WL), Waiting list with alternative intervention (WL-AI), Waiting list with usual alternative intervention (WL-UC). [d] At the end of the study, the control group may or may not receive the new intervention. (CBI)-Caregiver Burden Index; (BI)-Memory and Behavior Problem Checklist;(RMBPC)-Revised Memory and Behavior Problem Checklist;(MBPC)-The Memory and Behavior Problems Checklist;(CSS)-Caregiver Strain Scale;(ICS)-Impact of Caregiver Scale.

Supplementary Table S4. Workshop studies.

Author	NCDs	Scale	Intervention time	Intervention end (weeks)	Drop out	Nurse	Country	Relationship	Sex	Education mean (SD)	Age (SD)	Income	Trainer	Control group[c]	Final control group[d]
Ostwald, 1999 [63]	Combined	(RMBPC)[a](BI)[b]	7	20	20%	YES	USA	NO	0,67	NO	64,86 (13,6)	NO	COMBINED	WL	ALL
Gallagher-thompson, 2000 [64]	Combined	(ICS)[a]	10	12	24%	YES	USA	COMBINED	0,83	14,4 (2,1)	59,7 (10,7)	NO	COMBINED	WL	SINGLE
Bourgeois, 2002 [20]	Alzheimer	(CSS)[b]	13	24	33%	NO	USA	SPOUSE	0,54	NO	72,62 (6,56)	NO	THERAPIST	CG(UC)	NO DEFINE

Note. [a] Partial data. [b] Global data. [c] Control group without intervention (CG), Control group with alternative intervention (CG-AI), Control group with usual alternative intervention (CG-UC), Waiting list without intervention (WL), Waiting list with alternative intervention (WL-AI), Waiting list with usual alternative intervention (WL-UC). [d] At the end of the study, the control group may or may not receive the new intervention. (CBI)-Caregiver Burden Index; (BI)-Memory and Behavior Problem Checklist;(RMBPC)-Revised Memory and Behavior Problem Checklist;(MBPC)-The Memory and Behavior Problems Checklist;(CSS)-Caregiver Strain Scale;(ICS)-Impact of Caregiver Scale.

Supplementary Table S5. Group therapy studies.

Author	NCDs	Scale	Intervention time	Intervention end (weeks)	Drop out	Nurse	Country	Relationship	Sex	Education mean (SD)	Age (SD)	Income	Trainer	Control group[c]	Final control group[d]
Zarit, 1987 [45]	Combined	(BI)[b]	8	8	35%	NO	USA	COMBINED	0,63	14,38(2,69)	62 (12,75)	NO	PSYCOLOGIST	WL	SINGLE
Schiwiebert, 1994 [46]	Combined	(CBI)[a]	4	4	27%	NO	USA	CHILDREN	0,86	NO	63,5 (NO)	NO	PSYCOLOGIST	WL	ALL
Quayhangen, 2000 [33]	Combined	(BI)[a]	*8*	8	NO	YES	USA	SPOUSE	0,63	14,42 (0,3)	71,83 (0,8)	NO	COMBINED	WL	SINGLE
Hebert, 2003 [47]	Combined	(BI)[b] (RMBPC)[b]	16	16	18%	NO	CANADA	COMBINED	0,8	11,98 (4,08)	59,78 (12,86)	NO	PSYCOLOGIST	CG(AI)	UNDEFINED
Gonyea, 2006 [48]	Alzheimer	(BI)[b]	5	5	10%	NO	USA	COMBINED	0,67	NO	64,4 (13,8)	NO	THERAPIST	CG(AI)	UNDEFINED
Derotrou, 2010 [49]	Alzheimer	(BI)[b]	12	12	15%	NO	FRANCE	COMBINED	0,68	11 (4)	65 (12)	NO	COMBINED	WL(UC)	ALL
Pahlavanzadeh, 2011 [50]	Other dementias	(BI)[b]	5	5	NO	NO	IRAN	COMBINED	0,76	NO	44,72 (14,79)	NO	PSYCHIATRIST	CG	UNDEFINED
Wang, 2011 [51]	Other dementias	(CBI)[b]	16	16	0%	YES	CHINA	COMBINED	0,65	NO	40,63 (8,21)	NO	NURSE	CG	UNDEFINED
Canonici, 2012 [52]	Alzheimer	(BI)[b]	24	24	16%	NO	BRAZIL	NO	0,93	9,8 (4)	54,2 (11,7)	NO	OTHER TRAINER	CG(UC)	UNDEFINED
Cheng, 2014 [54]	Alzheimer	(BI)[b]	8	8	4%	NO	CHINA	COMBINED	0,88	NO	54,02 (8,77)	NO	PSYCOLOGIST	CG(AI)	UNDEFINED
Cheng, 2016 [53]	Alzheimer	(BI)[b]	8	8	2%	NO	CHINA	COMBINED	0,86	NO	55,16 (10,92)	NO	PSYCOLOGIST	CG(UC); CG(AI)	UNDEFINED
Kor, 2019 [55]	Other dementias	(BI)[b]	10	10	14%	NO	CHINA	COMBINED	0,83	NO	57,1 (10,6)	NO	COMBINED	CG(UC)	UNDEFINED

Note. [a] Partial data. [b] Global data. [c] Control group without intervention (CG), Control group with alternative intervention (CG-AI), Control group with usual alternative intervention (CG-UC), Waiting list without intervention (WL), Waiting list with alternative intervention (WL-AI), Waiting list with usual alternative intervention (WL-UC). [d] At the end of the study, the control group may or may not receive the new intervention. (CBI)-Caregiver Burden Index; (BI)-Memory and Behavior Problem Checklist;(RMBPC)-Revised Memory and Behavior Problem Checklist;(MBPC)-The Memory and Behavior Problems Checklist;(CSS)-Caregiver Strain Scale;(ICS)-Impact of Caregiver Scale.

Supplementary Table S6. Individual therapy studies.

Author	NCDs	Scale	Intervention time	Intervention end (weeks)	Drop out	Nurse	Country	Relationship	Sex	Education mean (SD)	Age (SD)	Income	Trainer	Control group[c]	Final control group[d]
Quayhangen, 1989 [31]	Alzheimer	(BI)[b]	16	32	20%	NO	USA	NO	0,37	14,14 (3,6)	66,18 (7,02)	NO	THERAPIST	CG	UNDEFINED
Teri, 1997 [32]	Combined	(BI)[a]	9	9	14%	NO	USA	COMBINED	0,69	14,2 (2,7)	66,9 (11)	NO	THERAPIST	WL; CG(UC)	UNDEFINED
Quayhangen, 2000 [33]	Combined	(BI)[a]	8	12	NO	YES	USA	SPOUSE	0,63	14,42 (0,3)	71,83 (0,8)	NO	COMBINED	WL	SINGLE
Onor, 2007 [34]	Alzheimer	(CBI)[b]	16	16	0%	NO	ITALY	NO	0,62	8 (3,38)	59 (7,54)	NO	PSYCHIATRIST	CG(UC)	UNDEFINED
Martin-Carrasco, 2009 [35]	Alzheimer	(BI)[b]	16	40	11%	UNDEFINED	SPAIN	COMBINED	0,68	NO	58,44 (13,9)	NO	COMBINED	CG(UC)	UNDEFINED
Oken, 2010 [36]	Other dementias	(RMBPC)[a]	8	8	13%	NO	USA	COMBINED	0,76	NO	64,9 (10,05)	NO	UNDEFINED	CG(AI)	UNDEFINED
Hirano, 2011 [37]	Other dementias	(BI)[b]	12	12	14%	NO	JAPAN	UNDEFINED	0,67	NO	73,7 (4,4)	NO	UNDEFINED	CG	UNDEFINED
Kurz, 2012 [38]	Alzheimer	(BI)[b]	12	36	15%	NO	GERMANY	COMBINED	0,70	NO	64,86 (13,21)	NO	THERAPIST	CG(UC)	UNDEFINED
Chen, 2014 [39]	Other dementias	(CBI)[b]	12	12	NO	YES	CHINA	COMBINED	0,67	9,24 (4,62)	54,94 (13,19)	NO	NURSE	CG(UC)	UNDEFINED
Cuc, 2017 [40]	Other dementias	(BI)[b]	1,42;6	24	18%	NO	USA	COMBINED	0,69	15,7 (2,6)	70,2 (12,5)	NO	UNDEFINED	CG(AI)	UNDEFINED
Deoliveira, 2018 [41]	Other dementias	(BI)[b]	24	24	NO	NO	BRAZIL	COMBINED	0,76	NO	58,7 (15,4)	NO	THERAPIST OCCUPATIONAL	CG(UC)	UNDEFINED
Gokugur, 2018 [43]	Other dementias	(BI)[b]	12	12	14%	YES	TURKEY	COMBINED	0,90	NO	52,52 (12,62)	NO	NURSE	CG(AI)	UNDEFINED
Tang, 2018 [43]	Other dementias	(BI)[b]	6	12	11%	NO	CHINA	COMBINED	0,79	13 (4,2)	57,1 (11,2)	NO	THERAPIST OCCUPATIONAL	CG(UC)	UNDEFINED

Tapark, 2019 [44]	Other dementias	(RMBPC) [a]	4	12	NO	NO	CHINA	COMBINED	0,86	NO	57,1 (11,1)	NO	UNDEFINED	CG(UC)	UNDEFINED

Note. [a] Partial data. [b] Global data. [c] Control group without intervention (CG), Control group with alternative intervention (CG-AI), Control group with usual alternative intervention (CG-UC), Waiting list without intervention (WL), Waiting list with alternative intervention (WL-AI), Waiting list with usual alternative intervention (WL-UC). [d] At the end of the study, the control group may or may not receive the new intervention. (CBI)-Caregiver Burden Index; (BI)-Memory and Behavior Problem Checklist;(RMBPC)-Revised Memory and Behavior Problem Checklist;(MBPC)-The Memory and Behavior Problems Checklist;(CSS)-Caregiver Strain Scale;(ICS)-Impact of Caregiver Scale.

Supplementary Table S7. Jadad scale quality studies.

Studies	J1	J2	J3	J4	J5	Scores (Jadad)
**Group therapy**						
Quayhangen, 2000 [33]	1	0	1	1	0	3
Canonici, 2012 [52]	1	0	0	0	1	2
Cheng, 2014 [54]	1	1	1	0	1	4
Cheng, 2016 [53]	1	1	1	1	1	5
Derotrou, 2010 [49]	1	1	0	0	1	3
Gonyea, 2006 [48]	1	1	0	0	1	3
Hebert, 2003 [47]	1	0	0	0	1	2
Kor, 2019 [55]	1	1	0	0	1	3
Pahlavanzadeh, 2011 [50]	1	0	0	0	0	1
Schiwiebert, 1994 [46]	1	0	0	0	1	2
Wang, 2011 [51]	1	1	0	0	1	3
Zarit, 1987 [45]	1	1	0	0	1	3
**Individual information**						
Dias, 2008 [29]	1	1	0	0	1	3
Gavrilova, 2009 [30]	1	1	0	0	1	3
McCurry, 2005 [28]	1	1	0	0	1	3
**Individual therapy**						

Quayhangen, 2000 [33]	1	0	1	1	0	3
Chen, 2014 [39]	1	0	0	0	0	1
Cuc, 2017 [40]	1	1	0	0	1	3
Deoliveira, 2018 [41]	1	1	1	0	0	3
Gokugur, 2018 [42]	1	1	0	0	1	3
Hirano, 2011 [37]	1	0	0	0	1	2
Kurz, 2012 [38]	1	0	0	0	1	2
Martin-Carrasco, 2009 [35]	1	1	0	0	1	3
Oken, 2010 [36]	1	1	0	0	1	3
Onor, 2007 [34]	1	0	0	0	1	2
Quayhangen, 1989 [31]	1	0	0	0	1	2
Tang, 2018 [43]	1	1	0	0	1	3
Tapark, 2019 [44]	1	1	0	0	0	2
Teri, 1997 [32]	1	0	0	0	1	2
**Respite care**						
Baumgarten, 2002 [56]	1	1	0	0	1	3
Mosello, 2008 [57]	1	0	0	0	1	2
Quayhangen, 2000 [33]	1	0	1	1	0	3
**Support group**						
Chien, 2008 [61]	1	0	0	0	1	2
Hebert, 1994 [58]	1	1	0	0	1	3
Logsdon, 2011 [62]	1	1	0	0	1	3
Roth, 2005 [60]	1	1	0	0	1	3
Yordi, 1994 [59]	1	0	0	0	1	2
Zarit, 1987 [45]	1	1	0	0	1	3
Teri, 1997 [32]	1	0	0	0	1	2

**Workshop**						
Ostwald, 1999 [63]	1	0	0	0	1	2
Bourgeois, 2002 [20]	1	0	0	0	1	2
Gallagher-thompson, 2000 [64]	1	0	0	0	1	2

Note. Higher score indicates higher quality.

Supplementary Table S8. Delphi list quality studies.

Studies	D1a	D1b	D2	D3	D4	D5	D7	D6	D8	Scores (Delphi list)
**Group therapy**										
Quayhangen, 2000 [33]	1	1	1	1	1	0	1	0	1	7
Canonici, 2012 [52]	1	0	1	1	0	0	1	0	1	5
Cheng, 2014 [54]	1	1	1	1	1	0	1	1	1	8
Cheng, 2016 [53]	1	1	1	1	0	0	1	1	1	7
Derotrou, 2010 [49]	1	1	1	1	0	0	1	1	1	7
Gonyea, 2006 [48]	1	0	1	1	0	0	1	0	1	5
Hebert, 2003 [47]	1	0	1	1	1	0	1	0	1	6
Kor, 2019 [55]	1	1	1	1	0	0	1	1	1	7
Pahlavanzadeh, 2011 [50]	1	0	1	1	0	0	1	0	1	5
Schiwiebert, 1994 [46]	1	1	1	1	0	0	1	0	1	6
Wang, 2011 [51]	1	1	1	1	1	0	1	0	1	7
Zarit, 1987 [45]	1	0	1	1	0	0	1	0	1	5
**Individual information**										
Dias, 2008 [29]	1	0	1	1	1	0	1	0	1	6
Gavrilova, 2009 [30]	1	0	1	1	1	0	1	1	1	7
McCurry, 2005 [28]	1	0	1	1	1	0	1	0	1	6
**Individual therapy**										
Quayhangen, 2000 [33]	1	1	1	1	1	0	1	0	1	7
Chen, 2014 [39]	1	0	1	1	0	0	1	0	1	5
Cuc, 2017 [40]	1	0	1	1	0	0	1	0	1	5
Deoliveira, 2018 [41]	1	1	1	1	1	1	1	1	1	9
Gokugur, 2018 [42]	1	0	1	1	0	0	1	1	1	6

Hirano, 2011 [37]	1	0	1	1	0	0	1	0	1	5
Kurz, 2012 [38]	1	1	1	1	1	0	1	0	1	7
Martin-Carrasco, 2009 [35]	1	0	1	1	0	0	1	0	1	5
Oken, 2010 [36]	1	1	1	1	1	0	1	0	1	7
Onor, 2007 [34]	1	0	1	1	0	0	0	0	1	4
Quayhangen, 1989 [31]	1	0	1	1	0	0	1	0	1	5
Tang, 2018 [43]	1	0	1	1	0	0	1	0	1	5
Tapark, 2019 [44]	1	0	1	1	0	0	1	0	1	5
Teri, 1997 [32]	1	1	1	1	1	0	1	0	1	7
**Respite care**										
Baumgarten, 2002 [56]	1	0	1	1	0	0	1	0	1	5
Mosello, 2008 [57]	1	0	1	1	0	0	1	0	1	5
Quayhangen, 2000 [33]	1	1	1	1	1	0	1	0	1	7
**Support group**										
Chien, 2008 [61]	1	0	1	1	1	0	0	0	1	5
Hebert, 1994 [58]	1	1	1	1	0	0	1	1	1	7
Logsdon, 2011 [62]	1	0	1	1	0	0	1	0	1	5
Roth, 2005 [60]	1	0	1	1	0	0	1	0	1	5
Yordi, 1994 [59]	1	0	1	1	0	0	1	0	1	5
Zarit, 1987 [45]	1	0	1	1	0	0	1	0	1	5
Teri, 1997 [32]	1	1	1	1	1	0	1	0	1	7
**Workshop**										
Ostwald, 1999 [63]	1	0	1	1	0	0	1	0	1	5
Bourgeois, 2002 [20]	1	0	1	1	0	1	1	0	1	6
Gallagher-thompson, 2000 [64]	1	1	1	1	1	0	1	0	1	7

Note. Higher score indicates higher quality.

Supplementary Table S9. Forbes scale quality studies.

Studies	F1	F2	F3	F4	F5	Scores (Forbes)
**Group therapy**						
Quayhangen, 2000 [33]	1	1	1	0	0	3
Canonici, 2012 [52]	1	1	1	0	1	4
Cheng, 2014 [54]	1	1	1	1	0	4
Cheng, 2016 [53]	1	1	1	1	0	4
Derotrou, 2010 [49]	1	1	1	0	1	4
Gonyea, 2006 [48]	0	1	1	1	0	3
Hebert, 2003 [47]	1	1	1	0	0	3
Kor, 2019 [55]	1	1	1	0	1	4
Pahlavanzadeh, 2011 [50]	1	1	1	0	0	3
Schiwiebert, 1994 [46]	1	1	1	0	0	3
Wang, 2011 [51]	1	1	1	1	0	4
Zarit, 1987 [45]	1	1	1	0	0	3
**Individual information**						
Dias, 2008 [29]	1	1	1	0	0	3
Gavrilova, 2009 [30]	1	1	1	0	0	3
McCurry, 2005 [28]	1	1	1	0	0	3
**Individual therapy**						
Quayhangen, 2000 [33]	1	1	1	0	0	3
Chen, 2014 [39]	1	1	1	0	1	4
Cuc, 2017 [40]	1	1	1	0	0	3
Deoliveira, 2018 [41]	1	1	1	0	0	3
Gokugur, 2018 [42]	1	1	1	0	0	3

Hirano, 2011 [37]	1	1	1	0	0	3
Kurz, 2012 [38]	1	1	1	0	0	3
Martin-Carrasco, 2009 [35]	1	1	1	0	0	3
Oken, 2010 [36]	1	1	1	0	0	3
Onor, 2007 [34]	1	1	1	1	1	5
Quayhangen, 1989 [31]	1	1	1	0	0	3
Tang, 2018 [43]	1	1	1	0	1	4
Tapark, 2019 [44]	1	1	1	0	0	3
Teri, 1997 [32]	1	1	1	0	0	3
**Respite care**						
Baumgarten, 2002 [56]	1	1	1	0	0	3
Mosello, 2008 [57]	1	1	1	1	1	5
Quayhangen, 2000 [33]	1	1	1	0	0	3
**Support group**						
Chien, 2008 [61]	1	1	1	1	1	5
Hebert, 1994 [58]	1	1	1	0	0	3
Logsdon, 2011 [62]	1	1	1	1	0	4
Roth, 2005 [60]	1	1	1	0	0	3
Yordi, 1994 [59]	1	1	1	0	0	3
Zarit, 1987 [45]	1	1	1	0	0	3
Teri, 1997 [32]	1	1	1	0	0	3
**Workshop**						
Ostwald, 1999 [63]	1	1	1	0	0	3
Bourgeois, 2002 [20]	1	1	1	0	0	3
Gallagher-thompson, 2000 [64]	1	1	1	0	0	3

Note. Higher score indicates higher quality.

Supplementary Table S10. Risk of bias (ROB) 2.0

Study ID	D1 - Randomisation process	D2 - Deviations from the intended interventions	D3 - Missing outcome data	D4 - Measurement of the outcome	D5 - Selection of the reported results	Overall
Group therapy						
Canonici, 2012 [52]	+	!	!	+	+	!
Cheng, 2014 [54]	+	!	+	+	+	!
Cheng, 2016 [53]	+	!	+	+	+	!
Derotrou, 2010 [49]	+	!	!	+	+	!
Gonyea, 2006 [48]	+	+	+	+	+	!
Hebert, 2003 [47]	+	!	!	+	+	!
Kor, 2019 [55]	+	!	!	+	+	!
Pahlavanzadeh, 2011 [50]	+	!	-	+	+	-

+ Low risk
! Some concerns
- High risk

Schiwiebert, 1994 [46]	+	+	!	+	+	!
Wang, 2011 [51]	+	!	+	+	+	!
Zarit, 1987 [45]	+	+	!	+	+	!
Quayhangen, 2000 [33]	+	!	-	+	+	-
Individual information						
Dias, 2008 [29]	+	!	!	+	+	!
Gavrilova, 2009 [30]	+	!	!	+	+	!
McCurry, 2005 [28]	+	!	!	+	+	!
Individual therapy						
Quayhangen, 2000 [33]	+	!	-	+	+	-
Chen, 2014 [39]	+	!	-	+	+	-
Cuc, 2017 [40]	+	!	!	+	+	!
Deoliveira, 2018 [41]	+	+	-	+	+	-
Gokugur, 2018 [42]	+	!	!	+	+	!
Hirano, 2011 [37]	+	!	!	+	+	!
Kurz, 2012 [38]	+	!	!	+	+	!

Martin-Carrasco, 2009 [35]	+	!	!	+	+	!
Oken, 2010 [36]	+	!	!	+	+	!
Onor, 2007 [34]	+	!	+	-	+	-
Quayhangen, 1989 [31]	+	+	!	+	+	!
Tang, 2018 [43]	+	!	!	+	+	!
Tapark, 2019 [44]	+	+	-	+	+	-
Teri, 1997 [32]	+	!	!	+	+	!
Respite care						
Baumgarten, 2002 [56]	+	!	!	+	+	!
Mosello, 2008 [57]	+	!	+	+	+	!
Quayhangen, 2000 [33]	+	!	-	+	+	-
Support group						
Teri, 1997 [32]	+	!	!	+	+	!
Chien, 2008 [61]	+	!	+	-	+	-
Hebert, 1994 [58]	+	!	!	+	+	!
Logsdon, 2011 [62]	+	!	+	+	+	!

Roth, 2005 [60]	+	!	!	+	+	!
Yordi, 1994 [59]	+	!	!	+	+	!
Zarit, 1987 [45]	+	+	!	+	+	!
Workshop						
Ostwald, 1999 [63]	+	!	!	+	+	!
Bourgeois, 2002 [20]	+	!	!	+	+	!
Gallagher-thompson, 2000 [64]	+	!	!	+	+	!

Supplementary Table S11. The PRISMA Checklist.

Section and Topic	Item #	Checklist item	Location where item is reported
**TITLE**			
Title	1	Identify the report as a systematic review.	Page 1
**ABSTRACT**			
Abstract	2	See the PRISMA 2020 for Abstracts checklist.	Page 2
**INTRODUCTION**			
Rationale	3	Describe the rationale for the review in the context of existing knowledge.	Page 3
Objectives	4	Provide an explicit statement of the objective(s) or question(s) the review addresses.	Page 3
**METHODS**			
Eligibility criteria	5	Specify the inclusion and exclusion criteria for the review and how studies were grouped for the syntheses.	Page 3
Information sources	6	Specify all databases, registers, websites, organisations, reference lists and other sources searched or consulted to identify studies. Specify the date when each source was last searched or consulted.	Page 3
Search strategy	7	Present the full search strategies for all databases, registers and websites, including any filters and limits used.	Page 3
Selection process	8	Specify the methods used to decide whether a study met the inclusion criteria of the review, including how many reviewers screened each record and each report retrieved, whether they worked independently, and if applicable, details of automation tools used in the process.	Page 3
Data collection process	9	Specify the methods used to collect data from reports, including how many reviewers collected data from each report, whether they worked independently, any processes for obtaining or confirming data from study investigators, and if applicable, details of automation tools used in the process.	Page 3
Data items	10a	List and define all outcomes for which data were sought. Specify whether all results that were compatible with each	Page 3-4

Section and Topic	Item #	Checklist item	Location where item is reported
		outcome domain in each study were sought (e.g. for all measures, time points, analyses), and if not, the methods used to decide which results to collect.	
	10b	List and define all other variables for which data were sought (e.g. participant and intervention characteristics, funding sources). Describe any assumptions made about any missing or unclear information.	Page 3-4
Study risk of bias assessment	11	Specify the methods used to assess risk of bias in the included studies, including details of the tool(s) used, how many reviewers assessed each study and whether they worked independently, and if applicable, details of automation tools used in the process.	Page 3-4
Effect measures	12	Specify for each outcome the effect measure(s) (e.g. risk ratio, mean difference) used in the synthesis or presentation of results.	Page 4
Synthesis methods	13a	Describe the processes used to decide which studies were eligible for each synthesis (e.g. tabulating the study intervention characteristics and comparing against the planned groups for each synthesis (item #5)).	Page 4
	13b	Describe any methods required to prepare the data for presentation or synthesis, such as handling of missing summary statistics, or data conversions.	Page 4
	13c	Describe any methods used to tabulate or visually display results of individual studies and syntheses.	
	13d	Describe any methods used to synthesize results and provide a rationale for the choice(s). If meta-analysis was performed, describe the model(s), method(s) to identify the presence and extent of statistical heterogeneity, and software package(s) used.	Page 4
	13e	Describe any methods used to explore possible causes of heterogeneity among study results (e.g. subgroup analysis, meta-regression).	Page 4
	13f	Describe any sensitivity analyses conducted to assess robustness of the synthesized results.	Page 4

Section and Topic	Item #	Checklist item	Location where item is reported
Reporting bias assessment	14	Describe any methods used to assess risk of bias due to missing results in a synthesis (arising from reporting biases).	Page 4
Certainty assessment	15	Describe any methods used to assess certainty (or confidence) in the body of evidence for an outcome.	Page 4
**RESULTS**			
Study selection	16a	Describe the results of the search and selection process, from the number of records identified in the search to the number of studies included in the review, ideally using a flow diagram.	Page 5
	16b	Cite studies that might appear to meet the inclusion criteria, but which were excluded, and explain why they were excluded.	Page 5
Study characteristics	17	Cite each included study and present its characteristics.	Page 5
Risk of bias in studies	18	Present assessments of risk of bias for each included study.	
Results of individual studies	19	For all outcomes, present, for each study: (a) summary statistics for each group (where appropriate) and (b) an effect estimate and its precision (e.g. confidence/credible interval), ideally using structured tables or plots.	Page 5
Results of syntheses	20a	For each synthesis, briefly summarise the characteristics and risk of bias among contributing studies.	Page 5-6
	20b	Present results of all statistical syntheses conducted. If meta-analysis was done, present for each the summary estimate and its precision (e.g. confidence/credible interval) and measures of statistical heterogeneity. If comparing groups, describe the direction of the effect.	Page 5-6
	20c	Present results of all investigations of possible causes of heterogeneity among study results.	Page 5-6
	20d	Present results of all sensitivity analyses conducted to assess the robustness of the synthesized results.	Page 5-6
Reporting biases	21	Present assessments of risk of bias due to missing results (arising from reporting biases) for each synthesis assessed.	Page 7

Section and Topic	Item #	Checklist item	Location where item is reported
Certainty of evidence	22	Present assessments of certainty (or confidence) in the body of evidence for each outcome assessed.	Page 7
**DISCUSSION**			
Discussion	23a	Provide a general interpretation of the results in the context of other evidence.	Page 7-9
	23b	Discuss any limitations of the evidence included in the review.	Page 9
	23c	Discuss any limitations of the review processes used.	Page 9
	23d	Discuss implications of the results for practice, policy, and future research.	Page 9
**OTHER INFORMATION**			
Registration and protocol	24a	Provide registration information for the review, including register name and registration number, or state that the review was not registered.	Page 2
	24b	Indicate where the review protocol can be accessed, or state that a protocol was not prepared.	Page 2
	24c	Describe and explain any amendments to information provided at registration or in the protocol.	Page 2
Support	25	Describe sources of financial or non-financial support for the review, and the role of the funders or sponsors in the review.	Page 10
Competing interests	26	Declare any competing interests of review authors.	Page 10
Availability of data, code and other materials	27	Report which of the following are publicly available and where they can be found: template data collection forms; data extracted from included studies; data used for all analyses; analytic code; any other materials used in the review.	Page 2

From: Page MJ, McKenzie JE, Bossuyt PM, Boutron I, Hoffmann TC, Mulrow CD, Shamseer L, Tetzlaff JM, Akl EA, Brennan SE, Chou R, Glanville J, Grimshaw JM, Hróbjartsson A, Lalu MM, Li T, Loder EW, Mayo-Wilson E, McDonald S, McGuinness LA, Stewart LA, Thomas J, Tricco AC, Welch VA, Whiting P, Moher D. The PRISMA 2020 statement: an updated guideline for reporting systematic reviews. Rev Esp Cardiol (Engl Ed). 2021 Sep;74(9):790-799. English, Spanish. doi: 10.1016/j.rec.2021.07.010. Erratum in: Rev Esp Cardiol (Engl Ed). 2022 Feb;75(2):192. PMID: 34446261.

Supplementary Figure S1. Heterogeneity evolution when excluding articles.

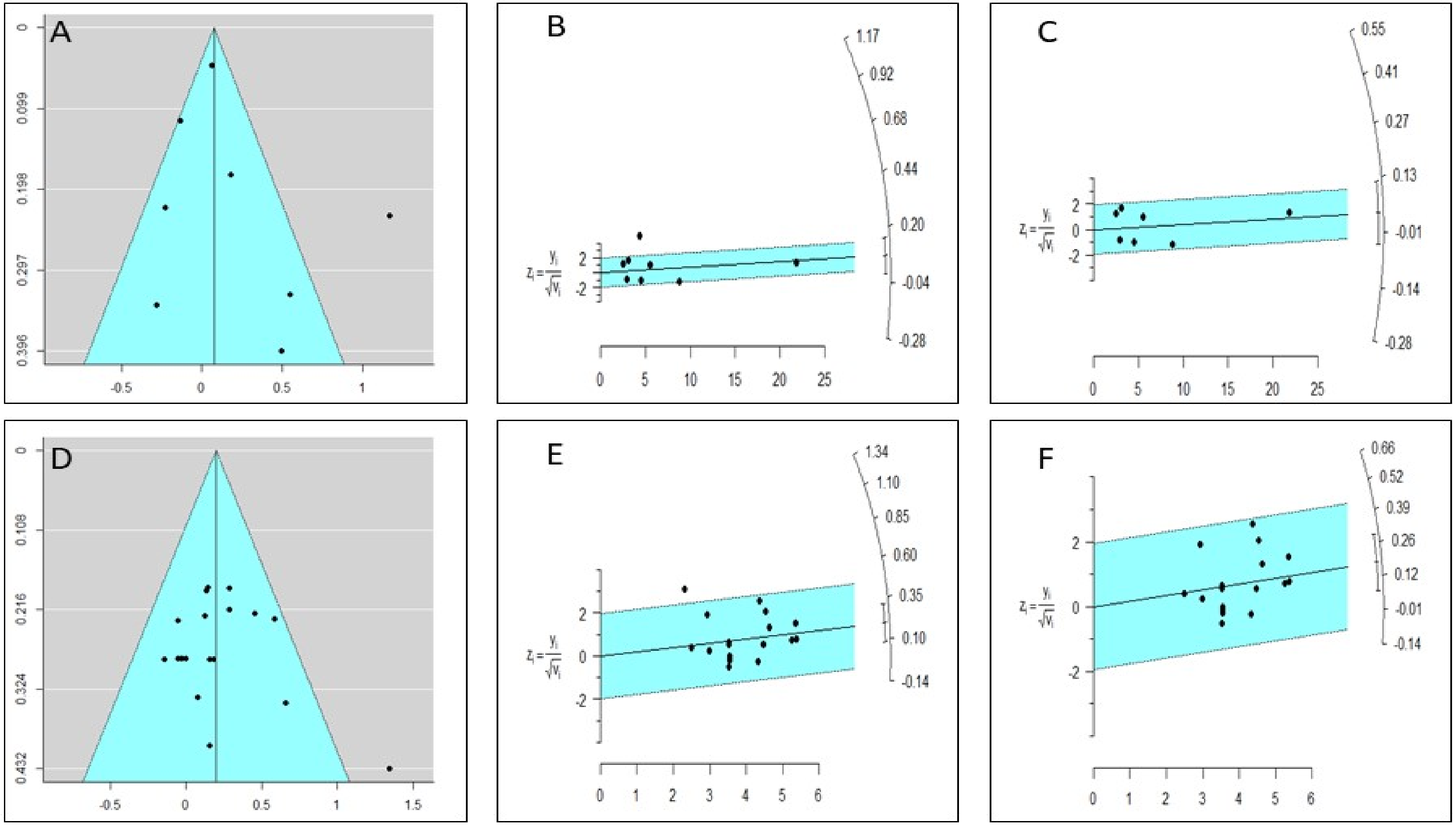

Note. Support group - A) Funnel plot; B) Galbriath plot; C) Galbriath plot without Chien et al. (2008). Group therapy - D) Funnel plot; E) Galbriath plot; F) Galbriath plot without Canonici et al. (2012).

Supplementary Figure S2. Heterogeneity evolution when excluding articles.

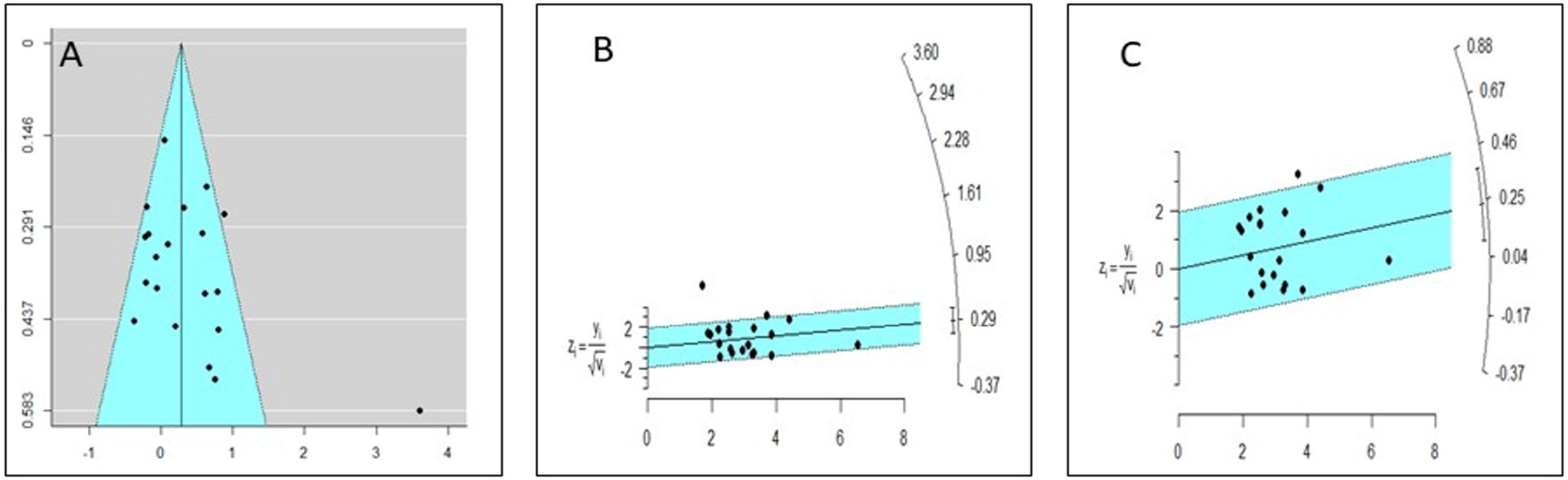

Note. Individual therapy - A) Funnel plot; B) Galbriath plot; C) Galbriath plot without Hirano et al. (2011).
